# Attention-deficit hyperactivity disorder (ADHD) stimulant medications as cognitive enhancers

**DOI:** 10.3389/fnins.2013.00082

**Published:** 2013-05-29

**Authors:** Claire Advokat, Mindy Scheithauer

**Affiliations:** Department of Psychology, Louisiana State UniversityBaton Rouge, LA, USA

**Keywords:** attention-deficit hyperactivity disorder, cognitive enhancement, episodic memory, stimulants, amphetamine, methylphenidate

## Abstract

Recent increases in attention deficit hyperactivity disorder (ADHD) diagnoses, and the escalation of stimulant prescriptions, has raised concern about diversion and abuse of stimulants, as well as the ethics of using these drugs as “cognitive enhancers.”Such concern appears misplaced in the face of substantial evidence that stimulant drugs *do not* improve the academic performance of ADHD-diagnosed students. Moreover, numerous studies have found little or no benefit of stimulants on neuropsychological tests of ADHD-diagnosed as well as normal, individuals. This paper examines the apparent paradox: why don't drugs that improve “attention,” produce better academic outcomes in ADHD-diagnosed students? We found that stimulant drugs significantly improved impairment of episodic memory in ADHD-diagnosed undergraduate students. Nevertheless, we also found consistent academic deficits between ADHD students and their non-ADHD counterparts, regardless of whether or not they used stimulant medications. We reviewed the current literature on the behavioral effects of stimulants, to try to find an explanation for these conflicting phenomena. Across a variety of behavioral tasks, stimulants have been shown to reduce emotional reactions to frustration, improve the ability to detect errors, and increase effortful behavior. However, all of these effects would presumably enhance academic performance. On the other hand, the drugs were also found to promote “risky behavior” and to *increase* susceptibility to environmental distraction. Such negative effects, including the use of drugs to promote wakefulness for last minute study, might explain the lack of academic benefit in the “real world,” despite their cognitive potential. Like many drugs, stimulants influence behavior in multiple ways, depending on the environmental contingencies. Depending on the circumstances, stimulants may, or may not, enhance cognition.

## Introduction

During the last few years the increase in non-medical use and misuse of the psychostimulant drugs methylphenidate and amphetamine, prescribed to treat Attention-Deficit/Hyperactivity Disorder (ADHD), has elicited much discussion and concern. One analysis of current use argues that the medical consumption of these two drugs is now comparable to past stimulant epidemics (Rasmussen, 2008), and implies that the increase parallels the increased rate of ADHD diagnoses among children and adults. In support of that interpretation, current best estimates for an ADHD diagnosis, of 5.9–7.1% in children and adolescents, and 5.0% in adults, represent 25% and 13.6% increases, respectively (Kessler et al., 2006; Willcutt, 2012; Getahun et al., 2013). Nearly 14 million monthly prescriptions for ADHD were written for Americans ages 20–39 in 2011, two and a half times the 5.6 million just 4 years before (Schwarz, 2013). In fact, between 1994 and 2009, a substantial increase in stimulant prescriptions occurred even *without* a clinical diagnosis of ADHD, or any other disorder (Olfson et al., 2013). Numerous surveys of college populations have reported an increase in stimulant prescriptions and a corresponding escalation of illicit use (Wilens et al., 2008; Advokat, 2010; Advokat and Vinci, 2012; Varga, 2012), with lifetime rates of diversion ranging from 5–29% (Wilens et al., 2008; Smith and Farah, 2011), although accurate data are difficult to obtain (Ragan et al., 2013).

Studies consistently show that the rationale among students for using stimulant medications, legally or not, is usually to improve academic performance, specifically to increase concentration, organization, and the ability to stay up longer and study (Advokat et al., 2008). Because these reasons for illicit use are not primarily recreational, it is not always considered to be as problematic as other types of drug abuse. Unfortunately, this is not necessarily the case, and the medical and legal consequences of illicit stimulant use may be underappreciated (Arria et al., 2008; Arria and DuPont, 2010).

Stimulant diversion and misuse have prompted considerable debate about the moral implications of using drugs to improve academic performance. Ethical discussions about taking drugs for “cognitive enhancement,” have been the subject of numerous editorials and commentaries (Farah et al., 2004; Greely et al., 2008; Harris, 2009). Thoughtful proposals for the responsible use of cognitive-enhancing drugs have called for the scientific study of the expected risks and the benefits to be gained as well as the moral consequences of allowing broad access to pharmacological enhancement of mental capacities.

One practical outcome was the decision of the Ethics, Law and Humanities Committee of the American Academy of Neurology (AAN) to release a special report, “Responding to requests from adult patients for neuroenhancements,” (Larriviere et al., 2009). According to lead author, Dan Larriviere, “A growing number of patients without illness believe they can improve their memory, cognitive focus and attention span by taking neuroenhancement drugs and are asking for prescriptions.” For the most part, these “neuroenhancers” consist of stimulant drugs. “The drugs most commonly used for cognitive enhancement at present are stimulants, namely Ritalin (methylphenidate) and Adderall (mixed amphetamine salts), and are prescribed mainly for the treatment of attention deficit hyperactivity disorder (ADHD).” This guideline concluded that physicians are allowed to grant requests for the stimulant drugs to improve cognition in healthy patients, although they are not obliged to do so. At the 60th Annual Conference of the Canadian Psychiatric Association in 2010 Dr. Derryck Smith presented a workshop on the subject and stated that psychiatrists should not hesitate to prescribe stimulants for neuroenhancement, if they wish.

The enthusiastic acceptance and embrace of stimulant-induced cognitive enhancement has not been universal. Outram (2010), Quednow (2010), and Lucke et al. (2011) argue that social demand for, and availability of, these drugs may not be as widespread as supposed, the efficacy of these drugs may be overrated, and their adverse psychological and physical effects may not be fully appreciated. Dr. Eric Racine and colleagues similarly argue against the prescription of medications for cognitive enhancement in healthy people (Forlini et al., 2012; Szalavitz, 2012; Schwarz, 2012, 2013).

## Clinical vs. “real world” effects of stimulants

Ever since amphetamine was discovered, serendipitously in the 1930's, to reduce hyperactivity in young boys (Baumeister et al., 2012) there is perhaps no other behavioral disorder than ADHD, for which drugs have so consistently proven acutely effective. Stimulants are an effective way of managing ADHD symptoms, such as short attention span, impulsive behavior, and hyperactivity. These drugs improve ADHD symptoms in about 70% of adults and 70–80% of children. They tend to reduce interruptive behavior, fidgeting, and other hyperactive symptoms.

But recent assessments of chronic benefit are less impressive. In fact, long-term completion rates of adults in clinical trials have been reported as low as 43–64% (Buitelaar et al., 2012b). In the first naturalistic study of ADHD-diagnosed adults treated with drugs for longer than 4 years, the score on a measure of mental health functioning was not different between those who were still on treatment and those who were no longer taking the medications (Lensing et al., 2013). Even in children, treatment discontinuation is high, with adherence typically ranging “from 36–84.8%” (Toomey et al., 2012, p. 763). The main reasons for discontinuation were “psychological side effects” (mood changes, irritability, depression, personality changes) and “perceived inadequate effectiveness.” Comparable results were reported in a review of medication adherence in adults with ADHD. While acknowledging the difficulty of accurately determining adherence rates, estimates ranged from 52–87% (Caisley and Müller, 2012). This was especially noteworthy because, as adults, the patients in these reports often took their medication “as needed,” and were not required to use the drugs every day. In a similar review of randomized clinical trials in adults, Castells et al. (2013) found the rate of all-cause treatment discontinuation of methylphenidate was not statistically different from placebo. This was in spite of the fact that methylphenidate was more efficacious than placebo in reducing symptoms.

In brief, even when the drugs are effective in reducing symptoms, ADHD patients often discontinue their stimulant medications. A recent study of this issue found that improvement in ADHD inattention and severity rating scores were not associated with functional improvement (Buitelaar et al., 2012a). In other words, symptom reduction does not always improve quality of life. As stated in one recent paper “While … results show that stimulants are effective in reducing symptoms of ADHD in adults, uncertainties remain as to their efficacy on other key aspects of the clinical picture” (Biederman et al., 2011, p. 509).

There is a similar disparity between the efficacy of stimulants, either amphetamine or methylphenidate formulations, for ADHD symptoms and their effect on cognitive performance. Reviews of this topic (de Jongh et al., 2008; Advokat, 2010; Repantis et al., 2010; Bidwell et al., 2011; Smith and Farah, 2011; Swanson et al., 2011; Advokat and Vinci, 2012) offer surprisingly little experimental support for stimulant-induced cognitive enhancement, not only in those with ADHD but also in normal individuals (Quednow, 2010). Articles in the New Yorker (Talbot, 2009) and Scientific American (Stix, 2009), describing the resurgence of these agents confirm the modest intellectual benefit derived from their “real world” use. The most recent review of long-term ADHD medication on academic outcomes in youth (Langberg and Becker, 2012) confirmed these earlier conclusions. As with all previous reviews, they found modest, but clinically unimpressive, improvement in several areas, including achievement test scores, grades, and grade retention. Ilieva et al. (2013) conducted a comprehensive investigation of the neuropsychological effects of mixed amphetamine salts in healthy, non-ADHD, young adults. They administered a battery of 13 tests, over 7 sessions, including standard memory assessments (face and word recognition), digit-span and n-back tests, tests of inhibitory control (Go/No-Go, Flanker task), of creativity (remote associations, embedded figures), standardized achievement tests, and a question about whether the participant felt that the drug influenced their performance. No cognitive outcome was enhanced (although, as often reported with stimulants, there was some slight improvement in those with lower baselines). Despite this objective outcome, participants believed their performance was more enhanced by the drug than by the placebo.

In brief, concern about the recent resurgence in illicit stimulant use and the ethics of stimulants as cognitive enhancers is ironic given the lack of evidence that they actually do enhance cognition. But this raises the question: *Why do drugs that acutely increase attention and concentration produce so little long-term intellectual benefit?*

### Effects of stimulant medications on cognitive measures in ADHD-diagnosed undergraduates

For several years, we have conducted studies in college undergraduates to address this question. In one set of experiments, neuropsychological tests were administered to control groups, (students who did not have ADHD) and to groups of students with ADHD who were tested when they were either “on” or “off” their medication. We used several types of tasks, ranging from motor dexterity, saccadic eye movements, attention, verbal fluency, memory (acquisition and retention of word lists), distractibility, and problem solving (Barrilleaux and Advokat, 2009; Advokat and Vinci, 2012).

In general, ADHD-diagnosed college students were not severely impaired on these tests relative to their normal peers. This was not surprising; all participants had been admitted to an accredited 4-year college. Because we did not do any assessments to confirm the diagnoses, it is reasonable to assume that some unknown proportion of the diagnoses were questionable. Even though we required that all participants provide a prescription for stimulants before they could be accepted into the study, we did not validate their diagnoses. On the other hand, the presumed cognitive effect of stimulants is not thought to be restricted to the ADHD population. Stimulants are also assumed to be capable of improving intellectual performance in cognitively normal individuals, although they usually are more effective if the baseline measure is below normal. In other words, we should have been able to detect some improvement even if the ADHD diagnoses were not valid. For that matter, it was also possible that some of the non-ADHD control group might have correctly been diagnosed with ADHD if they had been evaluated.

Our results can be summarized as follows: First, consistent with ADHD symptomatology, non-medicated ADHD students were more likely to be impaired on tasks that required some type of inhibitory reaction, in which they had to withhold their responses until the correct choice could be made (Barrilleaux and Advokat, 2009). Second, in most of our procedures, although performance was improved by stimulants, the amount of improvement was usually not significantly different from the normal, control, level. That is, ADHD-diagnosed students often did better on the neuropsychological tasks when they were on their medication, relative to when they were not. But, in most cases, neither result was different from the performance of normal students. Third, in those cases where the drugs did improve performance, the result was not due to the fact that stimulant drugs simply made the responses faster; it occurred whether the drug increased or decreased reaction times. It is not surprising that the most reliable effect of stimulants in our studies, as well as that of others (Advokat, 2010; Advokat and Vinci, 2012), is to reduce impulsivity, since that is one of the reasons they are prescribed. In fact, these drugs also reduce impulsivity in healthy adults (de Wit et al., 2002). But, a decrease in impulsivity would presumably be a beneficial effect; it would not explain why the drugs don't improve academic outcomes.

**Figure 1:**
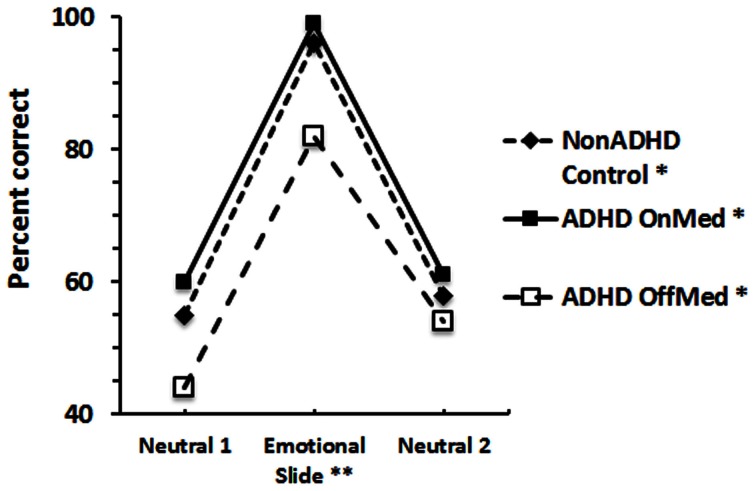
**Story recall of control and ADHD adults.** Average score of each group in each story phase. ^*^Significant difference within each group across the 3 story phases; ^**^Significant difference among the 3 groups on the Emotional Slide questions.

We then considered another alternative. Academic environments are inherently anxiety provoking, and arousal is known to affect memory. Modest levels can improve memory but too much anxiety will impair memory. This classic behavioral principle, known as the Yerkes-Dodson law, is often expressed in the form of an inverted inverted-U function. While it may not be supported in all types of situations, this relationship is more likely to be obtained with uncomplicated, simple, tasks. We hypothesized that perhaps adding a stimulating drug to the normal academic stresses of college produced too much arousal and impaired performance. To test this, we adapted a procedure developed by Cahill and McGaugh (1995) and used by Brignell et al. (2007), to study the effect of stimulants on episodic memory. The procedure consists of presenting a narrative slideshow that contains neutral passages and an emotional segment in the middle of the story. Participants watch the slides and listen to the story. Several days later they are asked questions about the narrative, to see how much they recall. Inevitably, information from the emotional segments is recalled more accurately than the neutral segments. We gave the same narrative slideshow to a group of normal students, and two groups of ADHD students, who were either on or off their medications. One week later all the students returned, under the same medication status, to answer questions about the story (Maul and Advokat, 2013). Figure 1 shows the percent of correctly answered questions in each group as a function of the story phase.

As expected, the control (non-ADHD), group recalled the “emotional” parts of the story significantly better than the “neutral” story segments. The data also show, for the first time, this “inverted-U shape” recall function in ADHD-diagnosed participants regardless of whether they were tested while on or off their medication. We appreciate that, in contrast to the classic definition, we cannot conclude that the descending limb of the U is due to a continued increase in arousal during the second neutral story segment. The outcome mainly illustrates an example of emotionally enhanced recall, which occurred reliably in all three groups.

In addition, the figure also shows that the performance of the non-medicated ADHD group was worse than the other groups on both, the first neutral segment, and on the emotional segment. Last, the score of the non-medicated group on the final segment was the same as that of the other two groups and was significantly better than on their first neutral phase.

These data show a substantial improvement, essentially normalization, of long-term, episodic, memory in medicated ADHD diagnosed adults. This result provides clear-cut evidence of cognitive benefit from stimulant drugs, and is one of the most dramatic examples of memory improvement demonstrated by stimulants. The results are reminiscent of Izquierdo et al. (2008), who found that subjects over 35–40 years old were significantly less likely to remember details about information they had learned 7 days before, relative to younger subjects. After methylphenidate, only the older subjects showed improvement; their age-related memory deficit was reversed, and they remembered as much information as the younger subjects. [Interestingly, episodic memory (of visual scenes) has also been improved in older adults (65–75 years old), when tested 6 h after receiving the dopamine precursor, levodopa (Chowdhury et al., 2012). Like stimulants, levodopa increases brain levels of the transmitter, dopamine].

In summary, our results show that stimulant medications *can* improve episodic memory in ADHD-diagnosed adults. Like the outcome of the neuropsychological assessments, the data suggest that the drugs do have some cognitive benefit. That is, in addition to their well-known ability to enhance attention, we found that stimulant drugs can also improve episodic recall. Again, however, this does not explain why they do not improve academic performance.

### Effects of stimulant medications on academic performance of ADHD-diagnosed undergraduates

Because they were college students, our presumptive ADHD population was perhaps not representative of the typical ADHD adult. Maybe they had learned how to gain some academic benefit from the stimulants. If that were true, they might not be academically impaired, relative to their non-ADHD counterparts. To find out more about the respective academic behavior of our undergraduates, we conducted a couple of surveys.

In the first study (Advokat et al., 2008) we surveyed three groups, ADHD-diagnosed students, non-ADHD, normal students, and a second group of non-ADHD, normal, students who acknowledged that they used the stimulants illicitly. First, we found that these three groups differed significantly in response to the question: “Do ADHD medications help academic performance.” A significantly greater proportion of the ADHD (and the normal/illicit use group), endorsed this statement compared with the normal group. This is consistent with the results reported by Ilieva et al. (2013), showing that their participants *believed* that stimulants improved their cognitive performance. Most important, the groups in our study also differed significantly in Grade Point Average (GPA), in that the GPA of the ADHD group (3.05, out of a possible 4.0) was significantly lower than that of the normal group (3.19).

Of course, we couldn't tell from these data how many students in the ADHD group actually used stimulant medications. Nor could we tell if the GPA would be even lower if those students didn't use the drugs. That is, we couldn't tell if the drugs were effective, either because they worked or because the students believed the drugs worked. To find out more about the influence of stimulant medications on academic outcome we next surveyed the self-reported study habits and strategies of ADHD-diagnosed and normal undergraduates (Advokat et al., 2011).

Our data showed that on average ADHD participants were diagnosed about 5 years before entering college. While nearly 98% of that group had initially taken ADHD medication, only 78.3% were currently using the drugs, while 19.6% stated that they were not (some respondents didn't answer all questions). When asked why they stopped taking stimulant medications, the majority cited side effects. Although specific side effects weren't always mentioned (“I did not like the way it made me feel; … make me feel crappy; the negative side effects outweigh the positives”), some individuals cited headaches, irritability, temporary heart rate elevation, nausea, sleep interference and “antisocial” feelings as examples of undesirable reactions. A few also stated that they either didn't need the drugs anymore, or, they wanted to see if they didn't need them anymore. Similar to the previous study, more than 90% of the students with ADHD endorsed the statement that medications helped them academically. Most of them stated that the drugs helped them to focus or to concentrate better, to pay attention, stay awake, and organize their studying. However, relatively few students (*n* = 6 out of more than 90) with ADHD specifically stated that they took medication to avoid distractions.

ADHD and non-ADHD undergraduates did not differ in their scores on the Scholastic Aptitude Test (SAT), Advanced Placement credits, scholarship awards, course credits, or number of hours they studied per week. Students in both groups believed they studied about the same as their peers, that the quality of their notes was the same, and stated that they rarely reviewed their class notes or read assigned readings before class.

However, the two groups did differ on several academic measures. As in our first study, the college GPAs of ADHD students were statistically lower than that of the Controls (2.94 vs. 3.12) and they also had significantly lower high school GPA and ACT scores (American College Testing is a standardized test for high school achievement and college admissions in the United States produced by ACT, Inc.). Although small, this difference was statistically significant, in spite of the fact that a minimum high school GPA and ACT score were required for university admission. ADHD students were also significantly more likely to withdraw from a class, to say that they were worse than other students at planning for and in completing class assignments, in their frequency of taking class notes, and in avoiding distractions.

One of the questions we asked in this survey was whether students studied “ahead of time” for exams, or whether they waited until just before the test to prepare. We then looked at how this related to their GPA. The results are shown in Table 1.

**Table 1:** **GPA as a function of study habits in ADHD and Non-ADHD undergraduates**.

	**When you study for an exam do you:**	
	**Non-ADHD (*n*)**	**ADHD (*n*)**
Study well before the exam?	3.12 (56)	3.16 (22)
Study in the day or two before the exam?	3.10 (86)	2.86 (64)
	ns	*p* < 0.05

The data in Table 1 show that the GPAs of non-ADHD students were the same whether or not they studied “ahead of time” for an exam. In other words, normal students did not “pay a price” in GPA for waiting until a few days before exams to study. In contrast, ADHD students who studied “well before” an exam had *higher* GPAs than those (the majority of this group) who didn't. Unlike normal students, ADHD students did “pay a price” if they waited until the last minute to study. Admittedly, this question did not distinguish among the possible types of exams (such as multiple choice or essay), nor did it take into account the level of knowledge that was assessed (such as application or synthesis). So we don't know if the students' preparation might differ depending on the nature of the assessments.

We then looked at whether the stimulant medications had any effect on the GPA of ADHD-diagnosed students as a function of whether they studied ahead of time or waited until the last minute. These data are shown in Table 2.

**Table 2:** **GPA of ADHD students: relationship between study habits and medication**.

**Study ahead of time?**	**Take medication?**	
	**Yes (*n*)**	**No (*n*)**
Yes	3.15 (19)	3.19 (3)
No	2.88 (47)	2.84 (15)

A two-way analysis of these GPAs found no effect of medication, but a statistically significant effect of study interval, (*F* = 4.06, *p* = 0.047). This result showed that, if ADHD students utilized the well-known strategy of studying ahead of time for exams, they could overcome their achievement deficit, even if they didn't take stimulant medications. In spite of the fact that only three ADHD students “studied ahead of time” without using the drugs, their GPA was comparable to that of the 19 undergraduates who did take the drugs in addition to using good study habits.

These data suggest that the GPA disparity between ADHD and non-ADHD students could be eliminated if ADHD students were able to develop well-established study habits. The results imply that the drugs alone are not sufficient to overcome the disadvantage of not preparing for exams. Unfortunately, it is not clear from these data alone if taking stimulant medications actually helps ADHD students to do that. That is, do the stimulant drugs help students to plan ahead, or to begin studying ahead of time so that they can compensate for their cognitive deficit? If so, why didn't more of the ADHD students do that?

It should be noted that even without good study habits, ADHD students were not failing. Their average GPA was just above a “C,” which means they were able to progress toward graduation at a normal pace. On the other hand, the absolute difference between the GPAs of ADHD students and normal students was numerically small. Even if the stimulants only increased energy and promoted wakefulness, it might be expected that they could bridge the gap. Why don't they?

## Non-cognitive behavioral effects of stimulant medications

Perhaps the problem lies with non-cognitive effects of stimulants. For example, these drugs are abused for their presumed euphoric effects. As McCloskey et al. (2010) point out, impulsiveness is often linked to abuse susceptibility; perhaps more impulsive individuals might experience greater euphoria from drugs, which might impair their ability to concentrate. They tested this by using a reaction time task, and comparing the subjective responses of normal adults, with their attention lapses, under amphetamine. In contrast to the prediction, participants with greater lapses of attention liked amphetamine *less* than those with fewer lapses, suggesting that ADHD students might be *less* affected by the euphoric properties of the stimulants than non-ADHD students.

What about other, non-cognitive, deficits in ADHD (Retz et al., 2012)? *Emotional dysregulation*, such as difficulty in dealing with stressful situations, excessive irritability and excitability, is also a symptom of the disorder. Emotional volatility may play a role, for example, in the increased risk of unsafe driving and traffic accidents in ADHD adults. While poor driving may be caused by inattention, it is also related to greater negative affect and increased frustration and anger in ADHD adults (Oliver et al., 2012). Studies of either simulated (Biederman et al., 2012) or real driving performance in ADHD adults (Cox et al., 2012) show significant improvement with stimulant drugs. Whether this occurs from increased attention or decreased frustration, or both, remains to be seen. But decreased frustration would presumably improve, or at least not impair, academic performance.

The ability to monitor and regulate one's actions is also thought to be impaired in ADHD. Hester and colleagues (2012) assessed this by adapting the standard “go/no-go” task, to make the inhibitory response more difficult and elicit more errors. In this procedure, participants indicate when they realize they made a mistake in responding. O'Connell et al. (2009) had previously found that ADHD participants were less likely than normal adults to recognize when they made an error. Hester et al. (2012) showed, in healthy adults that methylphenidate increased accuracy and significantly increased the proportion of errors that participants were aware of. Again, the direction of this drug effect would predict cognitive improvement with stimulants.

Yet another deficit associated with ADHD is a difficulty in sustaining effort. This is seen in the inability to complete projects and maintain academic or professional performance over long durations. To study this impairment deWit and colleagues developed an experimental protocol that required participants to choose whether to respond at a low rate to earn a low amount of money, or to respond at higher rates to earn more money. In either case, the probability of winning the money varied from high (88%) to medium (50%) to low (12%). In normal adults, amphetamine selectively increased the proportion of high rates of responding on the low probability trials. This was interpreted to mean that the stimulant selectively increased willingness to work harder, particularly when the probabilities are low (Wardle et al., 2011). Again, however, to the extent that this elegant paradigm is relevant to ADHD, the results would predict that stimulants should improve academic performance.

There are, however, some experimental paradigms in which stimulants worsen performance. Campbell-Meiklejohn et al. (2012a) devised a gambling game, in which participants had to choose, on each trial, whether they would risk a certain amount of money (the “stake”) after first starting out with a specified monetary loss. If the participant chose to play, and won, they would double their stake and previous losses were recovered. If they lost, the stake was lost and that amount was added to previous losses. The probability of winning varied, although this information was not given to the participants. Placebo players gambled less as the amount of the stake and the loss increased. However, participants given methylphenidate gambled at a consistent rate, which was above chance, and which remained the same across all stakes and trials. These results were similar to a study using rats (St. Onge et al., 2010) in which amphetamine also significantly appeared to increase “risky choice” compared to saline.

The data of Campbell-Meiklejohn et al. (2012a) were interpreted to mean that the stimulant “disrupted inhibitory influences on risky choice,” because participants who got methylphenidate did not reduce their gambling as the stakes and losses increased. However, these data might also suggest that stimulants impair “cognitive flexibility,” because behavior under methylphenidate did not seem responsive to changing contingencies. Impairment of cognitive flexibility, or “cognitive constriction,” was proposed many years ago as one cause of poor academic outcomes in stimulant-treated ADHD children (Advokat, 2010). A similar decrease in “cognitive flexibility,” might also help explain results of another study by Campbell-Meiklejohn and colleagues (2012b). In that experiment, methylphenidate, given to normal adults, appeared to promote behavior that conformed to “social norms.” Regardless of the interpretation, Campbell-Meiklejohn et al. (2012a) suggest, and we agree, that this type of drug effect might be relevant to the issue of stimulant-induced cognitive enhancement.

While the Campbell-Meiklejohn studies involved normal participants, Agay et al. (2010) directly compared adults with ADHD and healthy control participants on two versions of the Iowa Gambling Task. On the standard version, there was no effect of methylphenidate in either ADHD or non-ADHD participants. Each group made the same proportion of poor choices. On a modified version, ADHD participants made more risky choices than non-ADHD participants, which indicated a greater susceptibility to distraction. Oddly, methylphenidate had no effect on ADHD participants, but the drug *increased* the proportion of risky choices made by the non-ADHD, control, group. These data are consistent with the studies of St. Onge and Campbell-Meiklejohn, in showing that, in normal persons stimulants increase risky behavior (or promote inflexibility). But, because the stimulant did not improve the performance of ADHD participants, the authors speculated that, in environments that are very distracting, ADHD promotes poor decision-making and apparently the drugs don't help.

Results of a study by Prehn-Kristensen et al. (2011) also show that stimulants can worsen the effect of distractions. Although this study involved children (approximately 13–14 years old), and may not generalize to adults, the data show that adding a distracting stimulus to a working memory procedure *impaired performance only when the ADHD group was on medication*. Distracting stimuli did not reduce accuracy of either healthy controls or ADHD patients when they were off their medication.

## Summary and conclusions

Our research attempted to address the paradox of why drugs described as “cognitive enhancers” did not improve long-term academic performance of college students. Consistent with previous reports in adults with ADHD, we found minimal deficits on neuropsychological tests, and very modest improvements from stimulants. On the other hand, we discovered a substantial deficit in episodic memory in ADHD undergraduates, which was eliminated with stimulant medications. Whether or not this phenomenon would also occur in non-ADHD individuals remains to be seen. It might be that stimulant-induced facilitation of episodic memory will only occur when there is some baseline deficit, as is often the case with these drugs.

We also found the same academic impairment in ADHD college students previously reported in children and adolescents with the disorder. We further showed that in spite of the small absolute quantitative differences between these two groups, their deficits were not eliminated by stimulants alone. Moreover, our data show that, conversely, even without the stimulant drugs, ADHD undergraduates are capable of performing just as well in college as their non-ADHD peers, *if they acquire well-established effective study habits*. While these results increase our knowledge of ADHD related cognitive impairment, they do not explain why ADHD undergraduates, as a group, do not match their peers in academic performance.

To address that question we considered some non-cognitive behavioral effects of stimulants on mood and motivation. We found evidence that stimulants reduce frustration, improve self-regulation, and increase effortful behavior, and that the drugs' euphoric effects do not necessarily impair attention. However, all of these actions would facilitate academic performance and would not explain the discrepancy.

On the other hand, we also found evidence that stimulants “promote risky behavior” and may *increase* the interfering effect of environmental distractions. Results concerning risky behavior might be reinterpreted as evidence of stimulant-induced “inflexibility,” or “cognitive stereotopy,” which have been recognized for a long time (Robbins and Sahakian, 1979). Nevertheless, it may be that some students, either consciously or not, use these effects to their advantage. For example, the “inflexibility” that may be induced by stimulants might be put to good use by promoting a consistent, habitual, study schedule. Perhaps stimulant-induced facilitation of episodic memory does benefit ADHD students with good study habits. Alternatively, the drugs may not have much benefit if students use them to stay up longer the night before exams, or to write papers at the last minute. While we have not been able to definitively answer the question of why cognitive enhancers do not promote better academic outcomes, perhaps our results have helped reframe the issue for future research, so that policy decisions about substance abuse and cognitive enhancement might be better informed.

### Conflict of interest statement

The authors declare that the research was conducted in the absence of any commercial or financial relationships that could be construed as a potential conflict of interest.
